# Exosome-associated Shiga toxin 2 is released from cells and causes severe toxicity in mice

**DOI:** 10.1038/s41598-018-29128-9

**Published:** 2018-07-17

**Authors:** Miho Watanabe-Takahashi, Shinji Yamasaki, Masayuki Murata, Fumi Kano, Jun Motoyama, Jyoji Yamate, Jumpei Omi, Waka Sato, Hirofumi Ukai, Kentaro Shimasaki, Masaya Ikegawa, Miwa Tamura-Nakano, Ryohei Yanoshita, Yuri Nishino, Atsuo Miyazawa, Yasuhiro Natori, Noriko Toyama-Sorimachi, Kiyotaka Nishikawa

**Affiliations:** 1 Department of Molecular Life Sciences, Graduate School of Life and Medical Sciences, Doshisha University, Kyoto, Japan; 2 International Prevention of Epidemics, Graduate School of Life and Environmental Sciences, Osaka Prefecture University, Osaka, Japan; 3 Department of Life Sciences, Graduate School of Arts and Sciences, The University of Tokyo, Tokyo, Japan; 4 Cell Biology Center, Institute of Innovative Research, Tokyo Institute of Technology, Tokyo, Japan; 5 Laboratory of Developmental Neurobiology, Graduate School of Brain Sciences, Doshisha University, Kyoto, Japan; 6 Veterinary Pathology, Graduate School of Life and Environmental Sciences, Osaka Prefecture University, Osaka, Japan; 7 Genomics, Proteomics and Biomedical Functions, Graduate School of Life and Medical Sciences, Doshisha University, Kyoto, Japan; 8 Communal Laboratory, Research Institute, National Center for Global Health and Medicine, Tokyo, Japan; 9 Department of Pharmaceutical Sciences, Faculty of Pharmaceutical Sciences, Teikyo Heisei University, Tokyo, Japan; 10 Graduate School of Life Science, University of Hyogo, Hyogo, Japan; 11 Department of Health Chemistry, School of Pharmacy, Iwate Medical University, Iwate, Japan; 12 Department of Molecular Immunology and Inflammation, Research Institute, National Center for Global Health and Medicine, Tokyo, Japan

## Abstract

Shiga toxin (Stx), a major virulence factor of enterohemorrhagic *Escherichia coli* (EHEC), is classified into two subgroups, Stx1 and Stx2. Clinical data clearly indicate that Stx2 is associated with more severe toxicity than Stx1, but the molecular mechanism underlying this difference is not fully understood. Here, we found that after being incorporated into target cells, Stx2, can be transported by recycling endosomes, as well as via the regular retrograde transport pathway. However, transport via recycling endosome did not occur with Stx1. We also found that Stx2 is actively released from cells in a receptor-recognizing B-subunit dependent manner. Part of the released Stx2 is associated with microvesicles, including exosome markers (referred to as exo-Stx2), whose origin is in the multivesicular bodies that formed from late/recycling endosomes. Finally, intravenous administration of exo-Stx2 to mice causes more lethality and tissue damage, especially severe renal dysfunction and tubular epithelial cell damage, compared to a free form of Stx2. Thus, the formation of exo-Stx2 might contribute to the severity of Stx2 *in vivo*, suggesting new therapeutic strategies against EHEC infections.

## Introduction

Shiga toxin (Stx) is a major virulence factor of enterohemorrhagic *Escherichia coli* (EHEC) that causes bloody diarrhea and hemorrhagic colitis and exposure can result in fatal systemic complications, such as hemolytic uremic syndrome (HUS) and acute encephalopathy^1–4^. Stx produced by EHEC can be classified into two subgroups, Stx1 and Stx2, each of which is composed of subtypes that are closely related^5,6^. Stx1a and Stx2a are each a major subtype of Stx1 and Stx2, respectively, and show similar cytotoxicity in Vero cells and HeLa cells^7^; however, Stx2a is more toxic than Stx1a when injected into mice^8^ or primates^9–11^. The 50% lethal dose (LD_50_) of Stx2a in mice is approximately 400 times lower than that of Stx1a after intravenous or intraperitoneal administration^12^. Most importantly, epidemiological studies indicate that hemorrhagic colitis patients who are infected with EHEC that produce both Stx1 and Stx2 or produce Stx2 alone were more likely to develop serious complications, such as HUS, than patients infected with EHEC that produce only Stx1^13,14^. However, the molecular mechanism by which Stx2a induces more severe toxicity *in vivo* is not fully understood.

Stx molecules consist of a catalytic A-subunit, which has RNA *N*-glycosidase activity and inhibits eukaryotic protein synthesis^6,15^, and a B-subunit pentamer. The B-subunit pentamer is responsible for high-affinity binding to the functional Gb3 (Galα[1–4]-Galβ[1–4]-Glcβ-ceramide) receptors that are present on the plasma membrane surface of target cells^16,17^. After binding to Gb3, Stx is internalized into cells through endocytosis to form early endosomes and transported to the Golgi in a retrograde manner, and then to the endoplasmic reticulum (ER), from where the A-subunit is released into the cytosol to inhibit protein synthesis^18–21^. In this retrograde transport process, furin, a membrane-anchored protease that is primarily present in the trans-Golgi network (TGN), cleaves the A-subunit into an enzymatically active A1-fragment (27.5 kDa) and a carboxy terminal A2-fragment (4.5 kDa) that are linked by a disulfide bond and bind to the B-subunit, to be fully activated^22,23^. This disulfide bond is ultimately reduced in the ER lumen, liberating the A1-fragment to be released into the cytosol^24^.

Recent reports have shown that Stx1a and Stx2a have differences in intracellular transport. Although both toxins equally associated with lipid rafts on target cells where Gb3 is enriched, Stx2a was also found in detergent-soluble fractions (non-lipid rafts) of the membrane^25^. Furthermore, most of the internalized Stx2a co-localized with transferrin, which can function as a marker for a recycling endosome, but this was not the case with Stx1a^25^. These observations suggest that different endocytosis pathways may influence the intracellular transport of the toxins, even though both toxins are eventually transported to the Golgi after a 1-hr incubation with Vero cells^25^. Studies of the B-subunits of Stx1 and Stx2 using HeLa S3 cells transfected with Gb3 synthase show that both B-subunits are transported from early endosomes to the Golgi through a common pathway dependent on a surface-exposed area in the β4-β5 loop of each B-subunit^26^. However, the transport of the B-subunit of Stx2 to the Golgi occurred with slower kinetics compared to the B-subunit of Stx1^26^. Very recently, by using genome-wide siRNA screening, UNC50, a membrane protein present in the Golgi, was identified to specifically regulate the trafficking of Stx2 B-subunit, but not Stx1 B-subunit, from early endosomes to the Golgi^27^. Despite these observations, it is unclear whether differences in the intracellular transport of these toxins are related to the marked difference in toxin severity *in vivo*.

Recently, microvesicles (MVs), which are released into the extracellular space by many cell types, have received considerable attention because MVs play an essential role in mediating intercellular communication related to a wide variety of biological events, including immune responses, inflammation, and tumorigenesis^28–31^. MVs are composed of a lipid bilayer that contains transmembrane proteins, cellular cytosolic components, and nucleic acids. These structures are classified into two types of vesicles: shedding vesicles and exosomes, depending on their respective mechanism of generation and biochemical characteristics^28,29^. Shedding vesicles are released by direct budding of cytoplasmic contents from the plasma membrane, whereas exosomes are released by exocytic fusion of the external membrane of the multivesicular body (MVB) with the plasma membrane. In the plasma of patients with EHEC-associated HUS, microparticles that are produced by complement-activated leukocytes and platelets through an exogenous budding process are released in elevated levels^32,33^. Interestingly, some microparticles contain Stx2, resulting in the formation of pathogenic structures that have cytotoxic activity after incorporation via receptor independent endocytosis by target cells, such as glomerular endothelial cells^32^. However, microparticles can be also produced by Stx1 or by EHEC O157:H7-derived lipopolysaccharide (LPS) when incubated with whole blood cells^34^, suggesting that other structures or machineries might contribute to the difference in toxicity of Stx2 *in vivo*.

In this study, we found that after Stx2a was incorporated into target cells, the active form of the toxin with a cleaved A-subunit was actively released into the extracellular space via recycling endosomes, but that this was not the case with Stx1a. Part of the released Stx2 was associated with exosomes (referred to as exo-Stx2), which can cause severe toxicity and renal dysfunction in mice. Our observations indicate that critical differences in intracellular transport of Stx1 and Stx2 result in the formation of the unique structure of Stx2 that is related to more severe toxicity of Stx2 *in vivo*.

## Results

### Stx2a is actively released into the culture medium as an active form depending on its B-subunit

**Figure 1:**
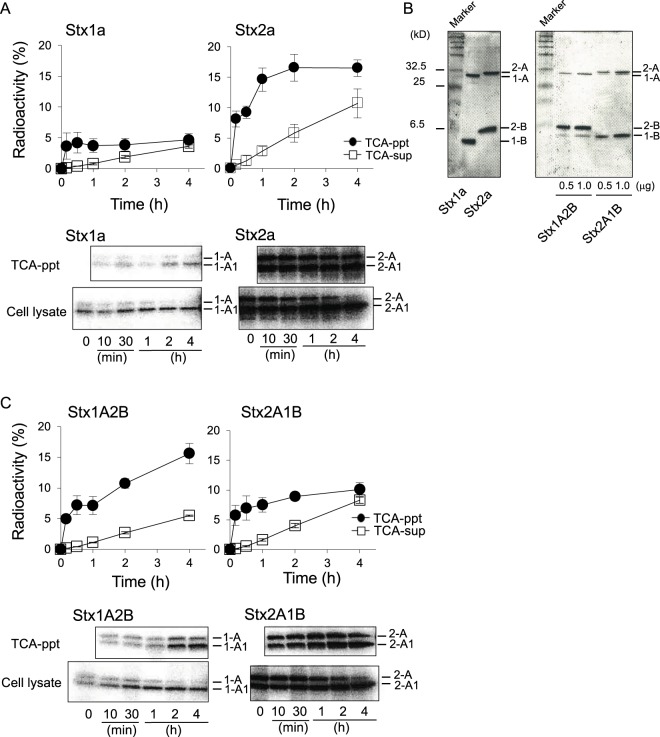
Stx2a is actively released into the culture medium depending on its B-subunit. (**A**) Vero cells were treated with 1 μg/ml of ^125^I- Stx1a or ^125^I- Stx2a for 2 hr at 37 °C, and then further cultured for 1 hr without Stx to incorporate the toxin. After washing, the cells were cultured for the indicated period. At each time point, cell lysates and TCA-ppt of the culture medium were prepared and then separated by electrophoresis and visualized (whole images are presented in Supplementary Fig. S1). Radioactivity of the TCA-sup, the TCA-ppt, and the cell lysates was measured by a γ-counter. The data are presented as the percentage of the total radioactivity (mean ± SE, *n* = 3). (**B**) Purified Stx1a (1 μg), Stx2a (1 μg), Stx1A2B, and Stx2A1B were analyzed by electrophoresis on an SDS/16% PAGE and visualized by CBB staining. (**C**) Vero cells were treated with 1 μg/ml of ^125^I-Stx1A2B or ^125^I- Stx2A1B for 2 hr at 37 °C and then further cultured another 1 hr without Stx. The metabolism of each Stx was analyzed as described in (**A**). Whole images are presented in Supplementary Fig. S2. The data are presented as the percentage of the total radioactivity (mean ± SE, *n* = 3).

After incorporation of ^125^I- Stx1a or ^125^I- Stx2a into Vero cells and the release of the toxin and its degradation products into the culture medium, the toxin and degradation products from the TCA-ppt and the TCA-sup were recovered and measured. As shown in Fig. 1A, Stx2a, but not Stx1a, was actively released in a time dependent manner with concomitant increase of the degradation products. In both cases, a major fraction of the released Stx A-subunit was found to be cleaved into an A1-fragment and an A2-fragment that are linked with a disulfide bond and bind to the B-subunit (Figs 1A and S1). This finding indicates that, before release, Stx is transported to the late/recycling endosomes and to the trans-Golgi network (TGN), where proteolytic cleavage by furin occurs^22^. After a 4-hr incubation, 27% of the total amount of Stx2a incorporated into the cells (TCA-ppt plus TCA-sup) was released, in contrast to Stx1a, of which less than 7% was released. Similar results were obtained with HeLa S3 cells and human renal proximal tubule cells (data not shown), suggesting that the active release of Stx2a is a common event.

To determine whether the active release of Stx2a was dependent on its B-subunit, we prepared a hybrid holotoxin composed of Stx1a A-subunit and Stx2a B-subunit (Stx1 A2B) and a hybrid holotoxin composed of Stx2a A-subunit and Stx1a B-subunit (Stx2A1B). The metabolism of each hybrid holotoxin was examined as described above. Hybrid toxin purity was confirmed by SDS-PAGE and only trace amounts of Stx1 B-subunit and Stx1 A-subunit were detected in purified Stx1A2B and Stx2A1B, respectively (Fig. 1B). After a 4-hr incubation, the Stx2A1B was recovered from the culture medium (TCA-ppt plus TCA-sup) and a radioactivity measurement was found to be 16% of the total amount incorporated into the cells, which was lower than the radioactivity measured from recovered Stx2a (27%; TCA-ppt plus TCA-sup) (Fig. 1A,C). In contrast, the radioactivity of Stx1A2B recovered from the culture medium was almost 3-fold that of Stx1a (7%). In both cases, a large amount of the toxin released into the culture medium was a nicked holotoxin (Fig. 1C). These observations clearly indicate that the active release of Stx2a and Stx1A2B results from the presence of the Stx2a B-subunit.

### Stx2a is preferentially transported to the recycling endosomes to be released into the culture medium in a Rab11 dependent manner

**Figure 2:**
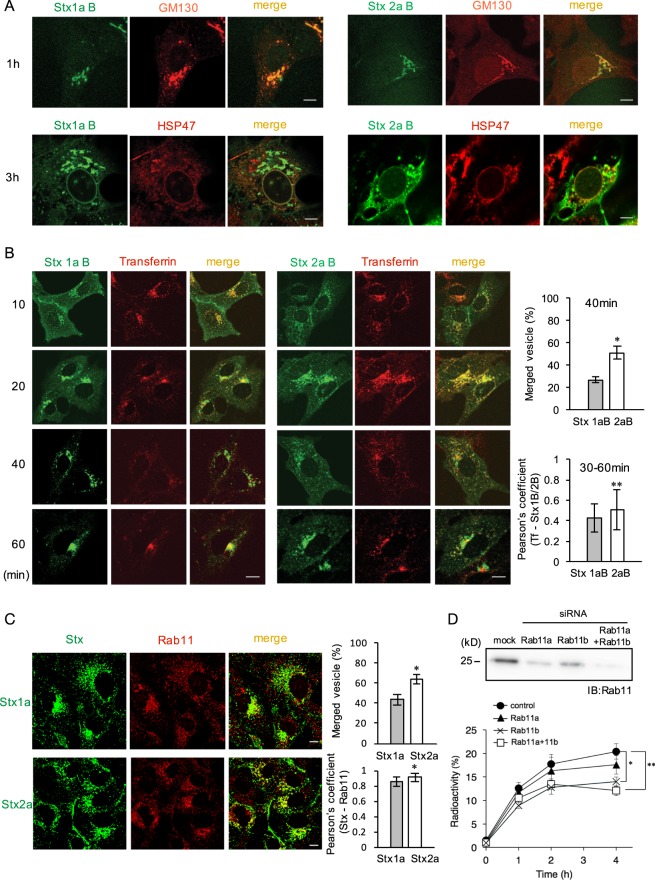
Stx2a is preferentially transported to the recycling endosomes to be released into the culture medium. (**A**) Vero cells were treated with Stx1a B-subunit or Stx2a B-subunit (10 μg/ml) for the indicated time periods at 37 °C. Each B-subunit was immunostained with anti-Stx1a or anti–Stx2a polyclonal antibody. Immunostaining of GM130 or HSP47 was performed after 1 hr or 3 hr incubation, respectively, using specific antibodies. Scale bars are 10 μm. (**B**) Vero cells were treated with Alexa Fluor 488-labeled Stx1a B-subunit or Stx2a B-subunit (10 μg/ml) in the presence of Alexa546-labeled transferrin (1 μg/ml) for 1 hr at 4 °C. After washing, cells were incubated at 37 °C for the indicated time period. Scale bars are 10 μm. The percentage of merged vesicles of the total number of Stx B-subunit positive vesicles in a single cell was measured after a 40 min incubation [right upper panel; mean ± SE, *n* (number of cells) = 17 or 7, for the Stx1a B-subunit or Stx2a B-subunit, respectively, from five independent experiments]. *P < 0.001 (Student’s *t*-test). Peason’s coefficient for co-localization between transferrin and Stx1a B-subunit or Stx2a B-subunit during 30–60 min is shown [right lower panel; mean ± SE, *n* (number of cells) = 49 or 37, for the Stx1a B-subunit or Stx2a B-subunit, respectively, from five independent experiments]. *P < 0.05 (Student’s *t*-test). (**C**) Vero cells were treated with Stx1a or Stx2a (1 μg/ml) for 1 hr at 37 °C. Immunostaining of Stxs and Rab11 were performed using specific antibodies. Scale bars are 10 μm. The percentage of merged vesicles of the total number of Stx positive vesicles in a single cell was measured [right upper panel; mean ± SE, *n (*number of cells) = 25 or 28, for Stx1a or Stx2a, respectively, from 2 independent experiments]. *P < 0.01 (Student’s *t*-test). Peason’s coefficient for co-localization between Rab11 and Stx1a or Stx2a is shown [right lower panel; mean ± SE, *n* = 46 or 49, for Stx1a or Stx2a, respectively, from five independent experiments]. *P < 0.01 (Student’s *t*-test). (**D**) Vero cells were transfected with 100 nM siRNA for Rab11a, Rab11b, or both Rab11s for 72 hr and the lysates were analyzed using anti-Rab11 antibody (upper panel). Whole images are presented in Supplementary Fig. S3. The transfected cells were treated with 1μg/ml of ^125^I- Stx2a for 2 hr at 37 °C, then further cultured another 1 hr without Stx. Cells were then washed and subsequently cultured for each indicated time period. Radioactivity present in the TCA-ppt of the culture medium was measured to quantify the amount of ^125^I- Stx2a released from the cells. The data are presented as the percentage of the total radioactivity (mean ± SE, *n* = 3). *P < 0.05; **P < 0.01 (Tukey’s HSD test).

To clarify the different roles of the Stx1a and Stx2a B-subunits in intracellular transport, we examined co-localization of each B-subunit with a Golgi maker (GM130) and an ER marker (HSP47), which can also be detected in the nuclear envelope, after treatment with each B-subunit in Vero cells. Both B-subunits co-localized with these markers, which indicates that after receptor-mediated endocytosis, each B-subunit is equally transported to the Golgi and then to the ER, as previously reported with holotoxins^21^ (Fig. 2A). To further investigate the differences in transport, we then focused on the recycling endosomes. In intracellular transport of Stx, the recycling endosomes are between the early endosomes and the Golgi^35,36^. We found that at an early stage, both B-subunits co-localized with transferrin, a recycling endosome marker. After 20 min, the co-localization of the Stx1a B-subunit with transferrin decreased in a time dependent manner, although about 50% of Stx2a B-subunit still co-localized with transferrin positive vesicles after 40 min (Fig. 2B), suggesting that the Stx2a B-subunit is more likely to stay in the recycling compartment.

Recently, it has been shown that the recycling route can be divided into two classifications: a rapid recycling route and a slow recycling route^37^. In both routes, the transferrin receptor can be recycled through^37,38^. Notably, the slow recycling route is involved in the transport of cargo proteins from the endocytic recycling compartment (ERC), which is molecularly defined by the presence of the small GTPase Rab11, to the plasma membrane^37^. Based on these observations, we examined the co-localization of each Stx with Rab11 after 1-hr incubation. Although both Stxs co-localized with Rab11, the merge ratio of Stx2a with Rab11 positive vesicles was higher than that of Stx1a with Rab11 positive vesicles (Fig. 2C), indicating that Stx2a is preferentially transported to the ERC, compared to Stx1a.

Rab11 has primarily been implicated in the control of vesicular trafficking from ERC to the plasma membrane and consists of three distinct subfamily members, Rab11a, Rab11b and Rab25^39,40^. Because Rab11a and Rab11b share 89% amino acid identity and are equally involved in the recycling of transferrin^39,41^, we examined the effects of knockdown of Rab11a, Rab11b, or both Rabs by using corresponding siRNAs, upon release of Stx2a into the culture medium. Efficient knockdown of each Rab11 family member was confirmed (Fig. 2D). The release of Stx2a into the culture medium was inhibited by 31% in Rab11b-knockdown and by 41% in Rab11a and Rab11b-knockdown cells, but less efficiently in Rab11a-knockdown cells (Fig. 2D), indicating that Rab11, especially Rab11b, is involved in the active release of Stx2a.

### A fraction of Stx2a released from the cells is associated with exosomes

**Figure 3:**
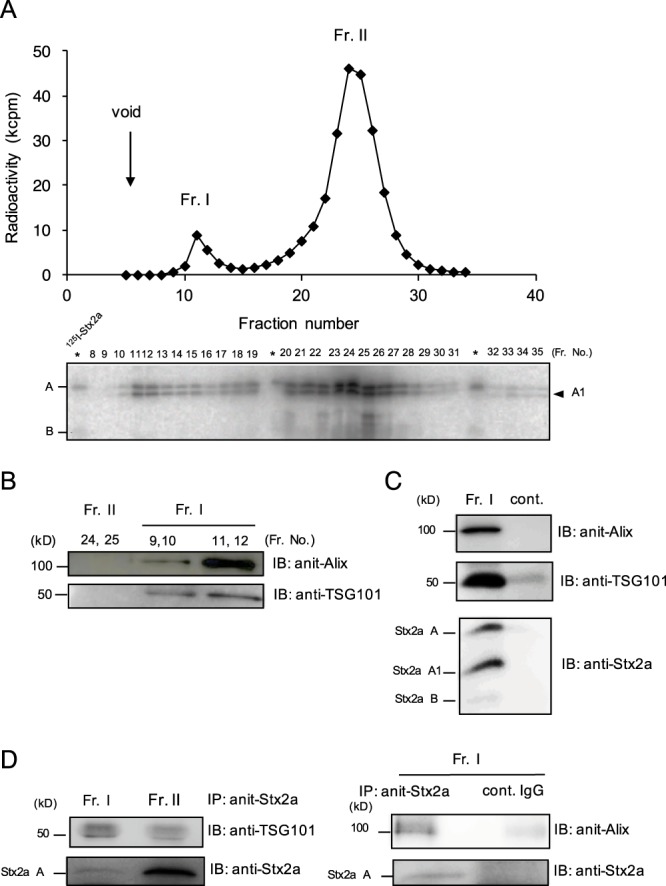
Fractionation and characterization of Stx2a released from HeLa S3 cells. (**A**) HeLa S3 cells were treated with 1 μg/ml of ^125^I- Stx2a for 2 hr at 37 °C, then further cultured another 1 hr without Stx. After washing, the cells were cultured for 6 hr, the cultured medium was centrifuged, and then the supernatant was separated by gel filtration column chromatography. Radioactivity present in each 1 ml fraction was measured and the ^125^I-Stx2a present in each fraction was separated by SDS-PAGE and visualized by autoradiography. Each asterisk indicates standard ^125^I- Stx2a. (**B**) The presence of Alix and TSG101 in Fr. I and Fr. II were analyzed by immunoblotting (whole images are presented in Supplementary Fig. S4A). (**C**) Alix, TSG101 and Stx2a present in exosomes prepared from Fr. I were detected by immunoblotting (whole images are presented in Supplementary Fig. S4B). As a negative control, all procedures were performed using the same amount of free Stx2a present in Fr. II (cont.). (**D**) Fr. I or Fr. II was immunoprecipitated with anti-Stx2a A-subunit monoclonal antibody and blotted with anti-TSG101 antibody or anti-Stx2 antibody (left panel). Fr. I was immunoprecipitated with anti-Stx2a A-subunit monoclonal antibody or control IgG and blotted with anti-Alix antibody or anti-Stx2 antibody (right panel). Whole images are presented in Supplementary Fig. S4C and D.

To characterize Stx2a that is released from the cells, the culture medium of HeLa S3 cells that were treated with ^125^I-Stx2a for 6 hr was recovered and separated on Sephacryl S-500 gel filtration column. Two peaks were detected, one of which corresponded to fractions numbered 10–15 (Fr. I) and the other to fractions numbered 17–28 (Fr. II) (Fig. 3A). Under the same conditions, original ^125^I-Stx2a was recovered in a single peak, corresponding to Fr. II (data not shown), indicating that the Fr. II contains a free form of Stx2a. In contrast, Fr. I, which was a higher molecular weight fraction, appeared to contain Stx2a associated with macromolecules, such as MVs, as judged by its retention volume^42^. After ultracentrifugation (100,000 × g, 2 hr, at 4 °C) of these fractions, over 60% of the ^125^I-Stx2a in Fr. I was recovered in the pellet, but the recovery from Fr. II was less than 2% (data not shown), providing further evidence for the presence of macromolecules containing Stx2a.

To characterize the macromolecule containing Stx2a, we used specific antibodies to detect the presence of two exosome markers, Alix and TSG101, in Fr. I. Both marker proteins, which are involved in MVB biogenesis^31^, were detected in Fr. I, but not in Fr. II, suggesting that Fr. I contained exosomes (Fig. 3B). After preparation of exosomes from Fr. I using ExoQuick exosome precipitation solution, the Stx2a A-subunit and cleaved A1-subunit were both detected in the exosome fraction along with Alix and TSG101, indicating that Stx2a in Fr. I was associated with exosomes (Fig. 3C). Furthermore, after immunoprecipitation of Stx2a in Fr. I with specific antibody for Stx2a A-subunit, both Alix and TSG101 were detected in the precipitate, confirming that this Stx2a is present on the surface of the exosomes containing Alix and TSG101 (Fig. 3D). In this work, we refer to the Stx2a present in Fr. I as exo-Stx2a and to the Stx2a in Fr. II as free-Stx2a.

**Figure 4:**
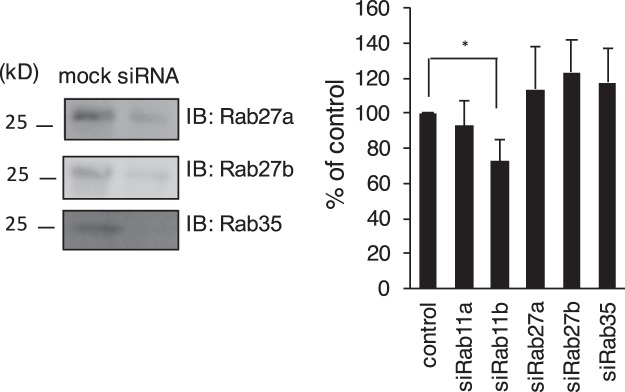
The effect of knockdown of Rab27a, Rab27b, or Rab35 on the release of Stx2a. Vero cells were transfected with 100 nM siRNA for Rab11a, Rab11b, Rab27a, Rab27b, or Rab35 for 72 hr and the lysates were analyzed using each anti-Rab antibody (left panel). Whole images are presented in Supplementary Fig. S5. The transfected cells were treated with 1μg/ml of ^125^I- Stx2a for 2 hr at 37 °C, then further cultured another 1 hr without Stx. Cells were then washed and subsequently cultured for 4 hr. Radioactivity present in the TCA-ppt of the culture medium was measured to quantify the amount of ^125^I- Stx2a released from the cells (right panel). The data are presented as the percentage of the total radioactivity (mean ± SE, *n* = 3). *P < 0.05 (Tukey’s HSD test).

In general, exosomes are released by exocytic fusion of the external membrane of the MVB with the plasma membrane^29,31^. We examined whether the origin of exo-Stx2a in the cells is from the MVB. Because it has been shown that specific Rab family members, such as Rab11^43^, Rab27a, Rab27b^44^ and Rab35^45^, are involved in the fusion and secretion steps in different types of cells^46^, we examined the effect of knockdown of these Rabs on the release of Stx2a in the culture medium. Under the condition that efficient knockdown of each Rab was confirmed, the release of Stx2a was efficiently inhibited by Rab11b-knockdown as described above, but not by the other Rabs-knockdown (Fig. 4), suggesting that exo-Stx2a is released by fusion of MVB with the plasma membrane in a Rab11b dependent machinery.

**Figure 5:**
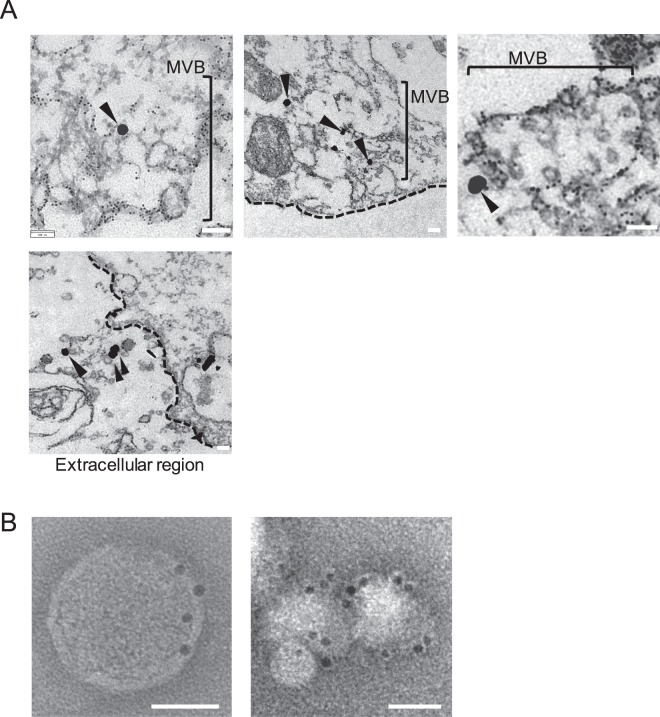
Immunoelectron microscopic analysis of ultrathin cryosections of cells treated with Stx2a and of exo-Stx2a in Fr. I. (**A**) Vero cells were treated with 1 μg/ml of Stx2a for 2 hr at 37 °C. After immobilization, the cells were first treated with anti-Stx2a polyclonal antibody, then treated with Alexa Fluor 488 FluoroNanogold-Fab’ labeled anti-rabbit IgG. The signal of Stx2a was enhanced by a gold enhancement method (black arrowheads). Dashed lines indicate cellular membrane. Scale bars are 100 nm. (**B**) Negative stain immunoelectron microscopic images of exo-Stx2a in Fr. I are shown. Fr. I prepared as described above was incubated with anti-Stx2A monoclonal antibody. Exosomes obtained from Fr. I were incubated with anti-mouse IgG conjugated to 10-nm gold particles. Scale bars are 50 nm.

Furthermore, we performed immunoelectron microscopic analysis. As shown in Fig. 5A, we could clearly detect the enhanced image of Stx2a, which was labeled with 1.4 nm Nanogold particles, in MVB structure and also on extracellular vesicles. We also performed negative stain immunoelectron microscopic analysis on exo-Stx2a in Fr. I. As shown in Fig. 5B, we could clearly detect the image of Stx2a on exosomes. All of these observations suggest that Stx2a can be released to the culture medium as free-Stx2a and exo-Stx2a through the formation of slow recycling endosomes and MVB, respectively, both of which are directly transported to the plasma membrane.

### exo-Stx2a is more toxic than free-Stx2a *in vivo*

**Figure 6:**
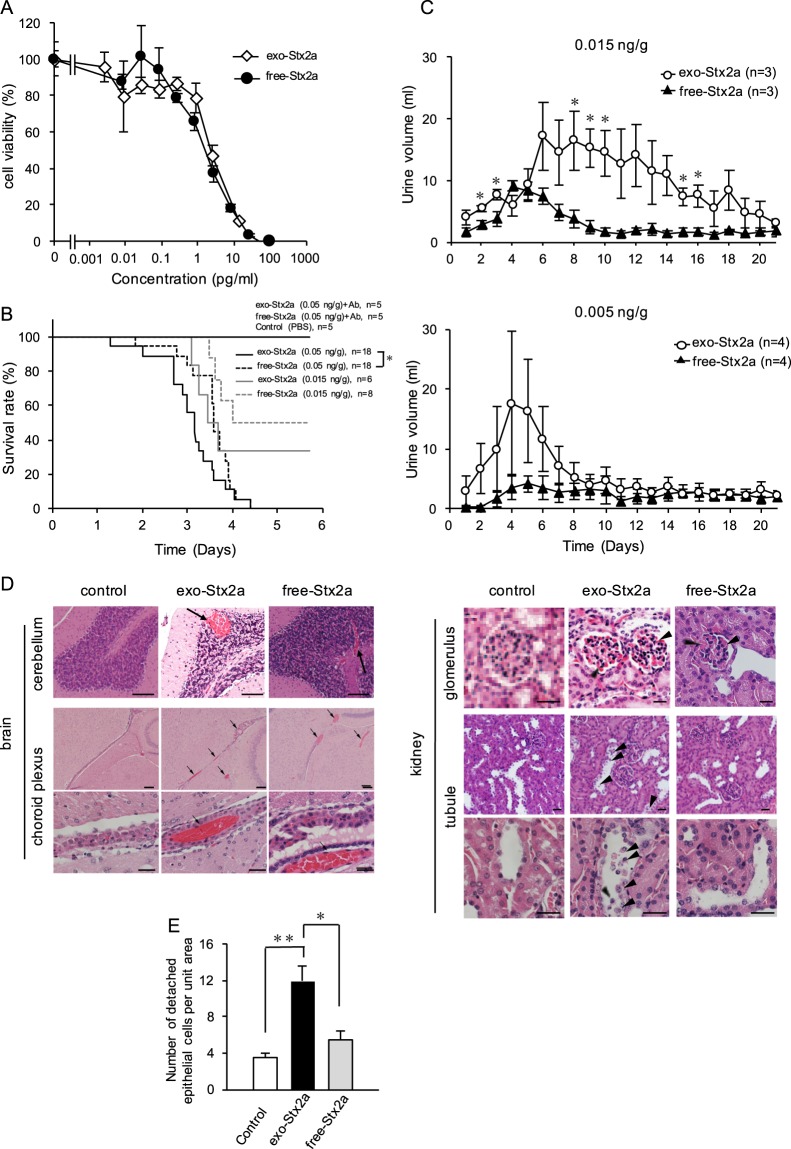
Comparison of the toxicity of exo-Stx2a and free-Stx2a *in vitro* and *in vivo*. (**A**) Cytotoxicity of exo-Stx2a or free-Stx2a, each of which was prepared by Sephacryl S-500 column chromatography as shown in Fig. 3, was examined in Vero cells. The data are presented as the percentage of viable cells in the absence of the toxin. Data from a representative experiment among three independent experiments are shown. (**B**) Indicated amount of exo-Stx2a or free-Stx2a (0.05 or 0.015 ng/g of body weight) was administered intravenously to the mice with or without anti-Stx2a B-subunit monoclonal antibody (2.5 μg/g of body weight). Data represent the survival rate of each group during the first 6 days after exposure. Control mice were treated with PBS. *P < 0.05 (Kaplan-Meier survival analysis and generalized Wilcoxon test, compared with 0.05 ng/g of free-Stx2a). (**C**) Indicated amount of exo-Stx2a or free-Stx2a (0.015 or 0.005 ng/g of body weight) was administered intravenously to the mice. The urine volume of each mouse on each day was measured (mean ± SE, *n* = 3–4). *P < 0.05 (Student’s *t* test). The urine volume of PBS treated control mice was 0.75 ± 0.16 ml/day (mean ± SE, *n* = 4) during the time periods. (**D**) exo-Stx2a or free-Stx2a (0.05 ng/g of body weight) was administered intravenously to the mice. After 3 days, sections of the brain and the kidney cortex were stained with hematoxylin and eosin. Arrows in the brain panels show congestion and hemorrhage. Arrowheads in the glomerulus panels show hyaline thrombus and disseminated intravascular coagulation. Arrowheads in the tubule panel show necrotic epithelial cells detached from the basement membrane. In the brain, scale bars in the upper, middle, and lower panels are 100, 100, and 20 μm, respectively. In the kidney, scale bars are 20 μm. (**E**) The number of detached epithelial cells in the tubules (**D**) per unit area (0.8 × 1 mm^2^) of each section was measured [mean ± SE, *n* (the number of unit area) = 10 from 2–4 independent experiments. *P < 0.005; **P < 0.001 (Student’s *t*-test).

We compared the toxicity of exo-Stx2a and free-Stx2a *in vitro* and *in vivo*. The cytotoxic activity of exo-Stx2a in Vero cells is the same as that of free-Stx2a (Fig. 6A). In addition, the relative abilities of each of these Stxs to inhibit protein synthesis, as measured by the uptake of [^3^H]Leu into cellular proteins, were almost the same in Vero cells and in HeLa S3 cells (data not shown). In contrast, the lethality of intravenously administered exo-Stx2a was higher than that of free-Stx2a at a dosage of 0.05 ng/g of body weight (*P* = 0.021, Generalized Wilcoxon test), but not at a dosage of 0.015 ng/g (*P* = 0.1, Generalized Wilcoxon test) (Fig. 6B). The average survival period of mice treated with 0.05 ng/g of exo-Stx2a or free-Stx2a was 3.1 ± 0.17 days and 3.5 ± 0.14 days, respectively. Co-administration of anti-Stx2a B-subunit monoclonal antibody completely rescued mice from the lethality of both toxins, demonstrating that the toxicity of exo-Stx2a can be attributed to the presence of Stx2a on the surface of the exosomes (Fig. 6B).

A marked increase of urine volume, an indication of renal dysfunction, was induced by treatment with exo-Stx2a (0.015 and 0.005 ng/g of body weight) and was also observed to a lesser extent after treatment with free-Srx2a (Fig. 6C). This result provides further evidence for the higher toxicity of exo-Stx2a *in vivo* compared to free-Stx2a. The marked increase of urine volume induced by the higher dose of exo-Stx2a (0.015 ng/g of body weight) was sustained up to 20 days after treatment (Fig. 6C).

We histologically examined tissue damage in mice treated with exo-Stx2a or free-Stx2a (0.05 ng/g of body weight). We noted pathological changes in the stratum granulosum cerebelli and the choroid plexus, including severe congestion and hemorrhage, equally in the brains from both exo-Stx2a treated and free-Stx2a treated mice (Fig. 6D). Additionally, we observed hyaline thrombus and disseminated intravascular coagulation in the renal glomeruli of the kidneys from both exo-Stx2a treated and free-Stx2a treated mice (Fig. 6D). In contrast, we found a marked increase of necrotic epithelial cells detached from the basement membrane in the tubules of the kidneys of exo-Stx2a treated mice, compared to free-Stx2a treated or control mice (Fig. 6D). This observation was supported by quantification of the detached epithelial cells in the tubules of these mice (Fig. 6E). Thus, exo-Stx2a can cause more severe damage against specific target cells, such as renal epithelial cells, than free-Stx2a. However, both exo-Stx2a and free-Stx2a cause damage to many parts of the brain and the kidney.

## Discussion

In this study, we found that Stx2a, but not Stx1a, is actively released after incorporation into target cells, in manner dependent on the B-subunit. Interestingly, some of the released Stx2a is present as exo-Stx2a, an exosome-associated form of Stx2a, which was we found caused more critical lethality and tissue damage in mice than free-Stx2a. Previously proposed reason for higher toxicity with Stx2a than Stx1a is an increased sensitivity of some types of cells, such as human renal microvascular endothelial cells^47^ and human brain microvascular endothelial cells^48^, to Stx2a than Stx1a. Our observations suggest a novel molecular mechanism, in which a unique structure containing activated Stx2a is formed, which is not formed by Stx1a, and this unique form contributes to the severe toxicity of Stx2a *in vivo*.

By comparing the metabolism of the hybrid toxins Stx1A2B and Stx2A1B, we showed that the active release of Stx2a depends on the presence of the Stx2a B-subunit. We further investigated the role of the B-subunit using purified B-subunit pentamers of Stx1a and Stx2a, both of which were shown to sufficiently hold the capacity to bind to the functional receptor Gb3 (data not shown). We found that the Stx2a B-subunit was more likely to stay in the recycling compartment prior to transport to the TGN, compared to the Stx1a B-subunit. All of these observations indicate that the active release of Stx2a occurs through the recycling route, and especially via the slow recycling route. We propose this model because the majority of released Stx2a was processed into A1- and A2-fragments by furin, which is present in the late/recycling endosomes and in the TGN^49,50^. The presence of this Stx2a specific transport pathway was further supported by the predominant co-localization of Stx2a with Rab11. Rab11 has been implicated to be involved in the slow recycling route, i.e. vesicular trafficking, from the ERC to the plasma membrane^37^. Additionally, the substantial inhibitory effect of Rab11 knockdown, especially Rab11b, on the release of Stx2a provides further evidence for our proposed model.

Rab11 consists of three distinct subfamily members: Rab11a, Rab11b, and Rab25^39,40^. Rab11a and Rab11b are equally involved in the recycling of many receptor proteins, including transferrin receptor^39,41^. However, our observation that the inhibitory effect of Rab11b knockdown on the release of Stx2a was greater than the effect of Rab11a knockdown and equal to the effect of a double knockdown clearly demonstrates that Rab11b is predominantly involved in the release of Stx2a through the slow recycling route. Only a few receptor proteins, including cystic fibrosis transmembrane conductance regulator^51^ and epithelial sodium channel^52^, have been shown to be recycled to the plasma membrane through the Rab11b specific pathway^51^, further emphasizing the uniqueness of the Stx2a specific transport pathway. On the other hand, a recent study has shown that Rab11a, Rab11b, and one of the Rab11 family interacting proteins (FIPs), FIP1/RCP, are all required for the retrograde transport of the Stx1a B-subunit from early/recycling endosomes to the TGN^53^. However, no information is available about the involvement of these molecules in the transport of the Stx2a B-subunit to the TGN or to the plasma membrane. Thus, a specific combination of Rab11b and its FIP(s) might be involved in Stx2a specific transport, although the precise molecular mechanism remains to be elucidated.

Here, we also found that exo-Stx2a contains the major exosome markers, Alix and TSG101, both of which are involved in MVB biogenesis^31^. This finding demonstrates that the origin of exo-Stx2a is in intraluminal vesicles (ILVs) formed during the maturation of MVB, which is thought to be derived from the ERC as described above. The immunoelectron microscopic analysis (Fig. 5) also supports this argument. Importantly, more than 10% of the Stx2a incorporated into cells was released into the culture medium as small degradation products, indicating that some part of the MVB with exo-Stx2a and free-Stx2a can be fused with the lysosome for degradation of the content before its fusion with the plasma membrane. In general, the involvement of MVB in the degradation of internalized materials is well-established^46,54^.

It has been shown that Rab11^43^, Rab27a, Rab27b^44^ and Rab35^45^, are involved in the fusion of MVB with the plasma membrane depending on cell types^46^. Among these Rabs, we found that only Rab11b is involved in the release of Stx2a. Previously, Evenness Interrupted (Evi), a Wingless-binding protein that is secreted along with Wingless at the neuromuscular junction, has been shown to be released in the form of exosomes from *Drosophila* Schneider-2 (S2) cells. The Evi-exosome release was inhibited by knockdown of Rab11, but not by knockdown of Rab27 or Rab35^55^, consisting with our results. In contrast, Lethal Factor of anthrax toxin has been shown to be incorporated into ILVs and released as exosomes in a manner dependent on Rab11 and Rab35, but not Rab27 in retinal pigment epithelial cells^56^. In HeLa cells, Rab27a and Rab27b were found to act in MVB docking at the plasma membrane to secrete exosomes^44^. All of these observations suggest that the involvement of these Rabs in the fusion and secretion steps depends on cell types and ingredients of exosomes.

Recently, it was reported that MVs, which are produced by complement-activated leukocytes and platelets through an exogenous budding process, are detected in elevated levels in the plasma of patients with EHEC-associated HUS and in the plasma of mice infected with Stx-producing EHEC^32–34^. Some of the platelet-, monocyte-, and neutrophil-derived MVs obtained from HUS patients or EHEC infected mice was found to contain Stx2^32^. However, the characteristics of these MVs are quite different from those of exo-Stx2a, in that the MVs, which bear C3 and C9 on the surface, can be equally produced by stimulating whole blood cells with Stx1, Stx2, or EHEC O157:H7-derived LPS^34^. Furthermore, without membrane permeabilization with saponin, no Stx2 was detected on the surface of the MVs, indicating that Stx2 is present inside the vesicles^32^. In contrast, exo-Stx2a was co-immunoprecipitated with Alix and TSG101, using an anti-Stx2a specific antibody, and co-administration of anti-Stx2a antibody completely rescued mice from the lethality of exo-Stx2a. These results strongly suggest that Stx2a is present on the surface of the exosomes. Thus, exo-Stx2a is a novel type of structure specific to Stx2a and its origin is from the exosomes, but not shedding vesicles.

We found that lethality of intravenously administered exo-Stx2a was higher than that of free-Stx2a, and that exo-Stx2a causes more severe renal dysfunction, as indicated by the marked increase of urine volume. Importantly, our histological data showed that the epithelial cells in the renal tubule are more susceptible to exo-Stx2a than free Stx2a, which does not cause substantial detachment of epithelial cells. We used 0.05 ng/g of body weight of Stx2a in these experiments. Previous reports have shown that administration of a higher dosage of Stx2a (0.08–0.225 ng/g of body weight) into mice intravenously or intraperitoneally causes severe damage of the tubules, including tubule dilation and necrotic and detached epithelial cells^12,57,58^. These observations suggest that tubular epithelial cells can be easily damaged by exo-Stx2a at a relatively low dosage, although damage by the free form of Stx2a occurs at a higher dosage. This conclusion is further supported by our finding that 0.015 ng/g of exo-Stx2a, but not free-Stx2a, causes a marked increase in urine volume, which is mainly regulated by tubular epithelial cell function. The precise mechanism by which exo-Stx2a exerts its severe toxicity *in vivo* is not clear. However, the higher sensitivity of some target cells, including tubular epithelial cells, against exo-Stx2a might provide a partial explanation for the severity of Stx2a *in vivo*. In conclusion, we have provided the first demonstration that the novel structure, exo-Stx2a, is more severely toxic in mice than free-Stx2a. This work suggests new avenues for therapeutic strategies, such as development of a compound that efficiently inhibits the production of exo-Stx2a *in vivo*.

## Materials and Methods

All methods were approved by Ethics Committees of Doshisha University, and all experiments were performed in accordance with approved protocol.

### Materials

Recombinant Stx1a and Stx2a were prepared according to published methods^19^. A hybrid holotoxin composed of Stx1a A-subunit and Stx2a B-subunit (Stx1A2B), a hybrid holotoxin composed of Stx2a A-subunit and Stx1a B-subunit (Stx2A1B), Stx1a B-subunit, and Stx2a B-subunit were prepared according to published methods^59^. Briefly, Stx1a or Stx2a was incubated in a dissociation solution (6 M urea, 0.1 M NaCl, 0.1 M propionic acid, pH 4) and each dissociated subunit was separated by gel filtration column chromatography (Sephacryl S-200; GE Healthcare Sciences, USA). The prepared Stx1a A-subunit and Stx2a A-subunit were mixed with heterologous B-subunit and dialyzed against 50 mM Tris-HCl (pH 7.4) to combine each subunit. Each combined hybrid holotoxin was further purified by gel filtration column chromatography (Sephacryl S-200). ^125^I-labeled Stx1a (^125^I-Stx1a), ^125^I-labeled Stx2a (^125^I-Stx2a), ^125^I-labeled Stx1A2B (^125^I-Stx1A2B), and ^125^I-labeled Stx2A1B (^125^I-Stx2A1B) were prepared by the iodine monochloride method as previously described^19^. Alexa Fluor 488-labeled Stx1a B-subunit and Stx2a B-subunit were prepared using the Alexa Fluor Protein Labeling Kit (Molecular Probes, USA).

### Metabolism of Stxs in Vero cells

Subconfluent Vero cells in a 24-well plate in Dulbecco’s modified Eagle medium (DMEM) supplemented with 10% fetal calf serum (FCS) were treated with 1 μg/ml of ^125^I- Stx1a, ^125^I- Stx2a, ^125^I- Stx1A2B, or ^125^I- Stx2A1B for 2 hr at 37 °C. After washing with Hank’s balanced salt solution, the cells were cultured with DMEM with FCS for 1 hr at 37 °C, washed, and then further cultured with DMEM without FCS for each indicated period. At each time point, after extensive washing, the cells were dissolved in lysis solution (0.1 M NaOH, 0.5% SDS) to prepare cell lysates. Cell viability was confirmed not to be affected at each time point. The cultured medium was recovered and treated with a final 10% trichloroacetic acid (TCA) for 1 hr at 4 °C. After centrifugation, the TCA-soluble supernatants (TCA-sup) were recovered as degradation products and the precipitated proteins were dissolved in lysis solution (TCA-ppt). The cell lysates and the TCA-ppt were separated by electrophoresis on an SDS/16% polyacrylamide gel (PAGE) and visualized by using a bio-imaging analyzer BAS-1000 (GE Healthcare Sciences, USA). Radioactivity present in the TCA-sup, the TCA-ppt, and the cell lysates was measured by a γ-counter (Perkin Elmer, Japan).

### Intracellular localization of Stx1a B-subunit, Stx2a B-subunit, Stx1a, and Stx2a in Vero cells

To examine co-localization of the Stx B-subunit with various organelle markers, subconfluent Vero cells in a glass dish (35 mm) were treated with Stx1a B-subunit or Stx2a B-subunit (10 μg/ml) for indicated time periods at 37 °C and fixed with 3% paraformaldehyde (PFA). Immunostaining of each B-subunit was carried out with rabbit anti-Stx1a or anti–Stx2a polyclonal antibody (obtained as described previously^20^) followed by Alexa Fluor 488-labeled goat anti-rabbit IgG (Invitrogen, Thermo Fisher Scientific Co., CA, USA). Immunostaining of GM130, a cis-Golgi marker, and HSP47, an ER marker, was performed using mouse anti-GM130 monoclonal antibody (BD Bioscience PharMingen, NJ, USA) and anti-Hsp47 monoclonal antibody (StressGen Bioreagents, BC, Canada) followed by Alexa Fluor 546-labeled goat anti-mouse IgG (Thermo Fisher Scientific Co.).

To examine co-localization of Stx B-subunits with a recycling endosome marker, transferrin, subconfluent Vero cells in a glass dish (35 mm) were treated with Alexa Fluor 488-labeled Stx1a B-subunit or Stx2a B-subunit (10 μg/ml) in the presence of Alexa546-labeled transferrin (1 μg/ml; Thermo Fisher Scientific Co.) for 1 hr at 37 °C. After washing, cells were incubated at 37 °C for the indicated period and analyzed by confocal laser scanning microscopy (Olympus, USA). To examine co-localization of Stxs with another recycling endosome marker, Rab11, Vero cells were treated with Stx1a or Stx2a (1 μg/ml) for 1 hr at 37 °C and fixed with 3% PFA. Immunostaining of Stx was carried out with rabbit anti-Stx1a or anti–Stx2a polyclonal antibody. Immunostaining for Rab11 was performed using mouse anti-Rab11 monoclonal antibody (Cell Signaling Technology, MA, USA) and Alexa Fluor 546-labeled goat anti-mouse IgG (Thermo Fisher Scientific Co.). All image analyses were performed using Image J software (NIH). Pearson’s coefficient was quantified with Coloc 2 plugin in Image J.

### RNA interference analysis

Knock down of Rab11a or Rab11b was performed using ON-TARGETplus SMARTpool siRNAs (Dharmacon, GE healthcare, CO, USA), each of which is comprised of 4 different siRNAs. Rab11a target sequences were 5′-GCAACAAUGUGGUUCCUAU-3′, 5′-CAAGAGCGAUAUCGAGCUA-3′, 5′-GUGCAGUGCUGUCAGAACA-3′, and 5′-GAGAUUUACCGCAUUGUUU-3′. Rab11b target sequences were 5′-UAACGUAGAGGAAGCAUUC-3′, 5′-GAGUACGACUACCUAUUCA-3′, 5′-UCGCCAAGCACCUGACCUA-3′, and 5′-CAACUUGUCCUUCAUCGAG-3′. Rab27a target sequences were 5′-CCAUAGCACUCGCAGAGAA-3′, 5′-CAGGAGAGGUUUCGUAGCU-3′, 5′-UCACAACAGUGGGCAUUGA-3′, and 5′-CUACGGAUCAGUUAAGUGA-3′. Rab27b target sequences were 5′-GGAACUGGCUGACAAAUAU-3′, 5′-GCAAAUGCUUAUUGUGAAA-3′, GGAAGUCAAUGAACGGCAA-3′, and 5′-UGAAACAAGUGCAGCAACU-3′. Rab35 target sequences were 5′-GAUGAUGUGUGCCGAAUAU-3′, 5′-CGAAGAACAGUAAACGAAA-3′, 5′-GAAGAUGCCUACAAAUUCG-3′, and 5′-GAAACGCUGCUGCUAAUGG-3′. For knockdown experiments, Vero cells were transfected with 100 nM siRNA (Dharmacon, GE healthcare) using Lipofectamine LTX (Invitrogen, Thermo Fisher Scientific Co.), according to the manufacturer’s protocol. Transfected cells were incubated 72 hr and analyzed by immunoblotting using anti-Rab11a antibody (Cell Signaling Technology), anti-Rab11b antibody (Cell Signaling Technology), or anti-Rab11 (Cell Signaling Technology) antibody. Using the transfected cells, the metabolism of ^125^I- Stx2a was examined as described above.

### Fractionation and characterization of Stx2a released from HeLa S3 cells

HeLa S3 cells in a 15 cm dish cultured in MEM supplemented with 10% FCS were treated with 1 μg/ml of ^125^I-Stx2a for 2 hr at 37 °C. After washing, the cells were cultured with MEM without FCS for 1 hr at 37 °C, then washed, and were further cultured with MEM without FCS for 6 hr. The culture medium was recovered and centrifuged at 10,000 × g. The supernatant was concentrated and then subjected to gel filtration column chromatography (Sephacryl S-500, 1 × 40 cm), which has a mean exclusion size of 200 nm and is suitable for fractionating macromolecules, equilibrated with 20 mM Tris-HCl (pH7.4) and 0.15 M NaCl, as described previously^42^. Radioactivity present in each fraction was measured by a γ-counter (Perkin Elmer). An aliquot of each fraction was separated by electrophoresis on an SDS/10 or 16% polyacrylamide gel and visualized by using a bio-imaging analyzer BAS-1000. The presence of Alix and TSG101 was analyzed by immunoblotting using anti-Alix antibody (Santa Cruz Biotechnology, Inc., TX, USA) and anti-TSG101 antibody (Santa Cruz Biotechnology, Inc.), respectively. Preparation of exosomes from the fractions with microvesicles was performed by using ExoQuick (System Biosciences Inc., CA, USA). The presence of Alix, TSG101, or ^125^I-Stx2a in the exosomes was also analyzed as described above. For the immunoprecipitation assay, culture medium prepared from ten dishes (15 cm) of HeLa S3 cells treated with 1 μg/ml of Stx2a and was separated by using Sephacryl S-500 as described above. The fractions with microvesicles were incubated with anti-Stx2a A-subunit monoclonal antibody (obtained from an originally prepared hybridoma) overnight at 4 °C and then treated with protein G agarose beads (GE healthcare) for 1 hr at 4 °C. After extensive washing, the bead fraction was separated by electrophoresis on an SDS/10 or 16% polyacrylamide gel and then analyzed by immunoblotting using anti-Alix antibody, anti-TSG101 antibody, or anti-Stx2 antibody.

### Immunoelectron microscopic analysis

Subconfluent Vero cells in a 100 mm dish in DMEM supplemented with 10% FCS were treated with 1 μg/ml of Stx2a for 2 hr at 37 °C. The cells were immobilized with 3% PFA for 30 min and treated with 25 μg/ml of Gigitonin (Wako Pure Chemical Industries, Osaka, Japan) for 30 min. The cells were treated with anti-Stx2a polyclonal antibody, followed with Alexa Fluor 488 FluoroNanogold-Fab’ anti-rabbit IgG (Nanoprobes Inc., NY, USA). The cells were post-fixed with Osmic acid solutions (2% OsO_4_, 0.32% K_3_[Fe(CN)_6_], 1% PFA, 1.25% glutaraldehyde in 30 mM HEPES buffer, pH7.4) for 1 hr at room temperature and embedded in Epon (Wako Pure Chemical Industries). The blocks were sectioned to 70 nm thickness with an ultramicrotome (EM UC7, Leica, Austria) and stained with uranyl acetate and lead citrate. Gold enhance EM plus (Nanoprobes, Inc.) was used according to manufacture’s instructions to enhance the image of the gold particles. The ultra-thin sections were examined with an electron microscope (JEM-1400, JEOL, Japan).

For negative stain immunoelectron microscopic analysis on exo-Stx2a, exosome-containing fractions (Fr. I), which were obtained by gel filtration column chromatography as described above, were incubated with anti-Stx2A monoclonal antibody for 1 h at room temperature. Preparation of exosomes from the fractions was performed by using ExoQuick (System Biosciences Inc.) as described above. The obtained fraction was incubated with anti-mouse IgG conjugated to 10-nm gold particles (BBI solutions, Crumlin, UK) for 1 h at room temperature. After washing, the fraction was applied to a glow-discharged carbon-coated copper grid (Nisshin EM, Co., Ltd., Tokyo, Japan) and stained with 2% uranyl acetate. The grid was examined with an electron microscope (JEM-2100, JEOL).

### Cytotoxicity assay

Subconfluent Vero cells cultured in a 96-well plate were treated with indicated amounts of free form of ^125^I-Stx2a or ^125^I-Stx2a associated with microvesicles, both of which were prepared from HeLa S3 cell culture medium using Sephacryl S-500 as described above, for 72 hr at 37 °C. The amount of each ^125^I- Stx2a was calculated based on its specific radioactivity. The relative number of living cells remaining after treatment was determined using a Cell Counting Kit-8 (Dojindo Laboratories, Kumamoto, Japan), as described previously^19^.

### Toxicity of Stx2a in mice

The indicated amount of free form ^125^I-Stx2a or ^125^I-Stx2a associated with microvesicles was prepared using Sephacryl S-500 column chromatography and was administered to female ICR mice (18–20 g, Japan SLC, Japan) with or without 2.5 μg of anti-Stx2s B-subunit monoclonal antibody through a tail vein and the survival periods of the mice were monitored. The data were analyzed by Kaplan-Meier survival analysis. To examine the effect of Stxs on urine volume, each mouse was kept in a metabolic cage and the urine sample was collected on each day. All animal experiments were approved by the animal ethics committee of Doshisha University prior to their commencement, and performed in accordance with approved protocol.

### Histological examination

Free form ^125^I-Stx2a or ^125^I-Stx2a associated with microvesicles (0.05 ng/g of body weight) was prepared as described above and was administered intravenously to female ICR mice (*n* = 2–4). After 3 days, the brains and the kidneys were immediately fixed in 10% formalin. The tissue was then embedded in paraffin, sectioned, and stained with hematoxylin and eosin.

### Statistical analysis

Significant differences among multiple groups were analyzed using one-way analysis of variance (ANOVA) followed by Tukey-Kramer post hoc tests. Significant differences between two groups were analyzed using one-way ANOVA followed by Student’s *t* test. Significant differences of survival rate were analyzed using Kaplan-Meier survival analysis and Generalized Wilcoxon test.

## Electronic supplementary material

Supplementary information
