# Supplementary material for: Exosome-associated Shiga toxin 2 is released from cells and causes severe toxicity in mice
Source: Sci Rep. 2018 Jul 17;8:10776. doi: 10.1038/s41598-018-29128-9 (PMC6050230; doi:10.1038/s41598-018-29128-9)
Supplement: Supplementary file 1 — Supplementary information [file 41598_2018_29128_MOESM1_ESM.pdf]

# Supplementary Information

# Exosome-associated Shiga toxin 2 is released from cells and causes severe toxicity in mice.

**Miho Watanabe-Takahashi[a], Shinji Yamasaki[b], Masayuki Murata[c], Fumi Kano[d], Jun Motoyama[e], Jyoji Yamate[f], Jumpei Omi[a], Waka Sato[a], Hirofumi Ukai[a], Kentaro Shimasaki[a], Masaya Ikegawa[g], Miwa Tamura-Nakano[h], Ryohei Yanoshita[i], Yuri Nishino[j], Atsuo Miyazawa[j], Yasuhiro Natori[k], Noriko Toyama-Sorimachi[l], and Kiyotaka Nishikawa[a]**

[a]Department of Molecular Life Sciences, Graduate School of Life and Medical Sciences, Doshisha University, Kyoto, Japan; [b]International Prevention of Epidemics, Graduate School of Life and Environmental Sciences, Osaka Prefecture University, Osaka, Japan; [c]Department of Life Sciences, Graduate School of Arts and Sciences, The University of Tokyo, Tokyo, Japan; [d]Cell Biology Center, Institute of Innovative Research, Tokyo Institute of Technology, Tokyo, Japan; [e]Laboratory of Developmental Neurobiology, Graduate School of Brain Sciences, Doshisha University, Kyoto, Japan; [f]Veterinary Pathology, Graduate School of Life and Environmental Sciences, Osaka Prefecture University, Osaka, Japan; [g]Genomics, Proteomics and Biomedical Functions, Graduate School of Life and Medical Sciences, Doshisha University, Kyoto, Japan; [h] Communal Laboratory, Research Institute, National Center for Global Health and Medicine, Tokyo, Japan ; [i]Department of Pharmaceutical Sciences, Faculty of Pharmaceutical Sciences, Teikyo Heisei University, Tokyo, Japan; [j]Graduate School of Life Science, University of Hyogo, Hyogo, Japan; [k]Department of Health Chemistry, School of Pharmacy, Iwate Medical University, Iwate, Japan; [l]Department of Molecular Immunology and Inflammation, Research Institute, National Center for Global Health and Medicine, Tokyo, Japan.

Correspondence and requests for materials should be addressed to K.N. (email: knishika@mail.doshisha.ac.jp)

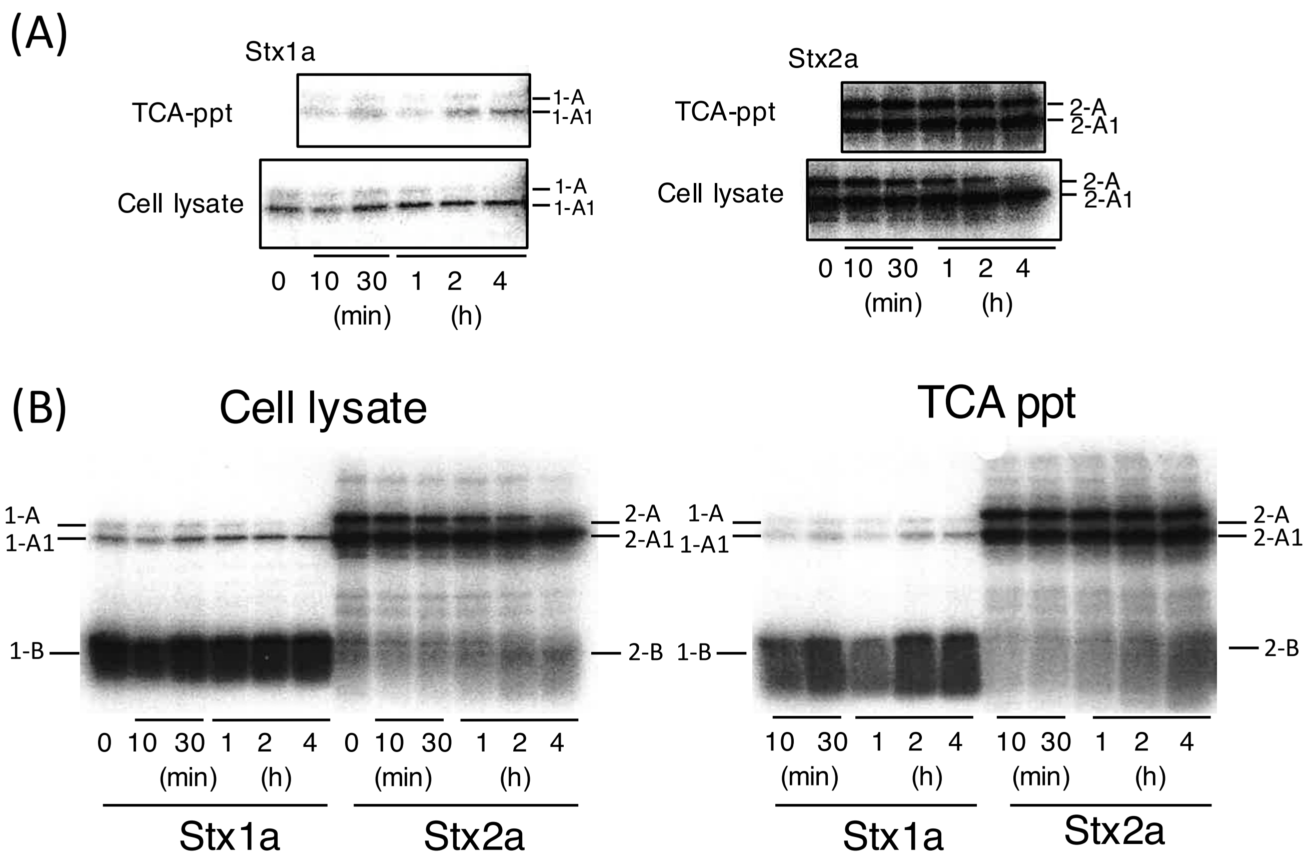


**Supplementary Figure S1.** Images presented in (A) were cropped from the whole images shown in (B).

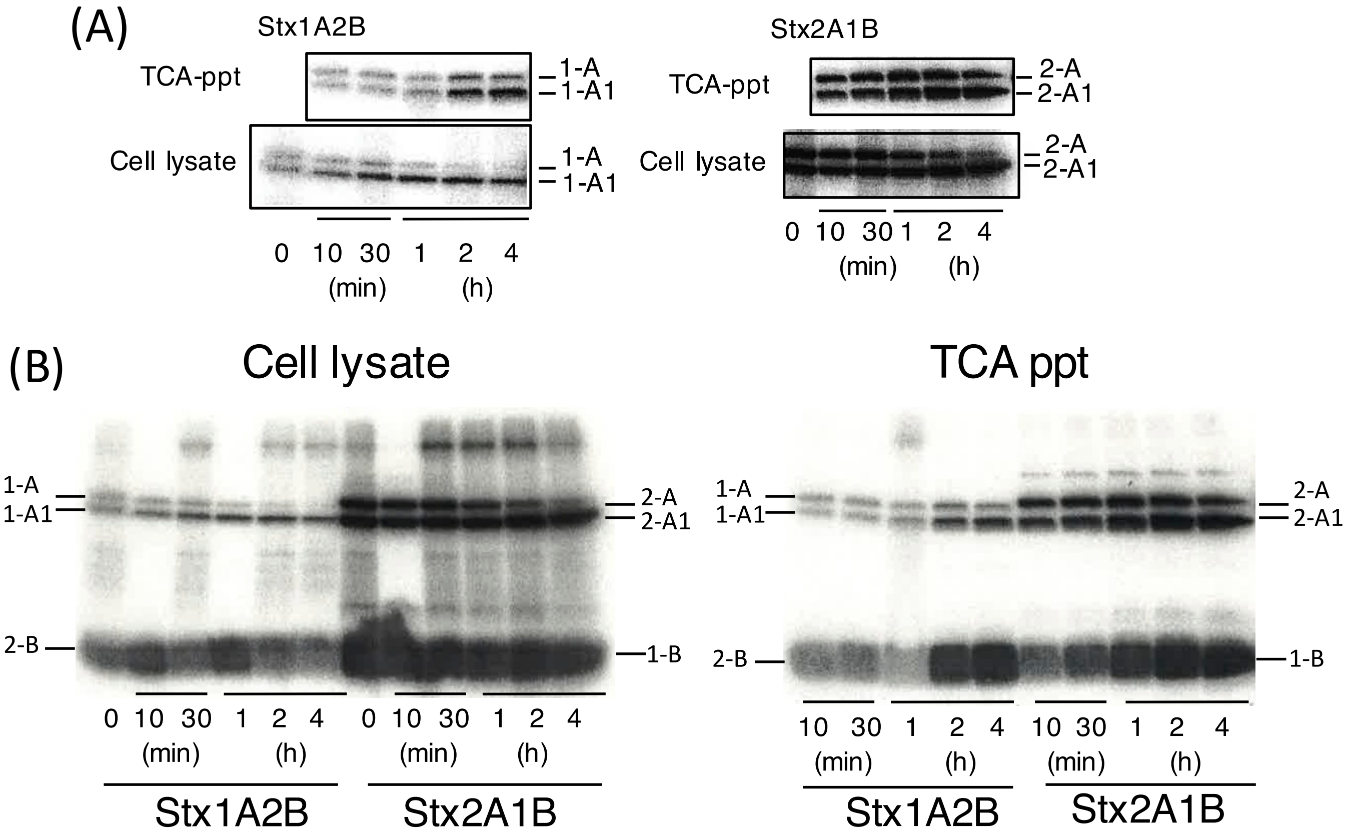


**Supplementary Figure S2.** Images presented in (A) were cropped from the whole images shown in (B).

(A)

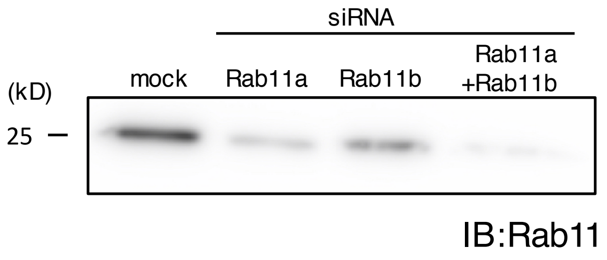


(B)

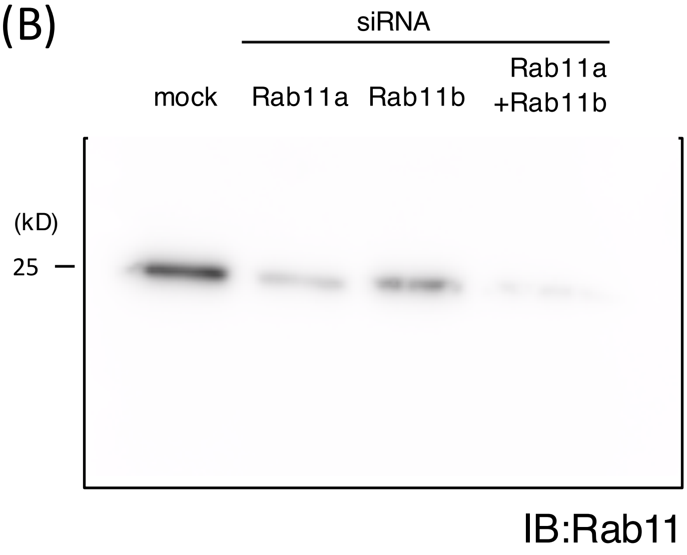


**Supplementary Figure S3.**

The image presented in (A) was cropped from the whole image shown in (B).

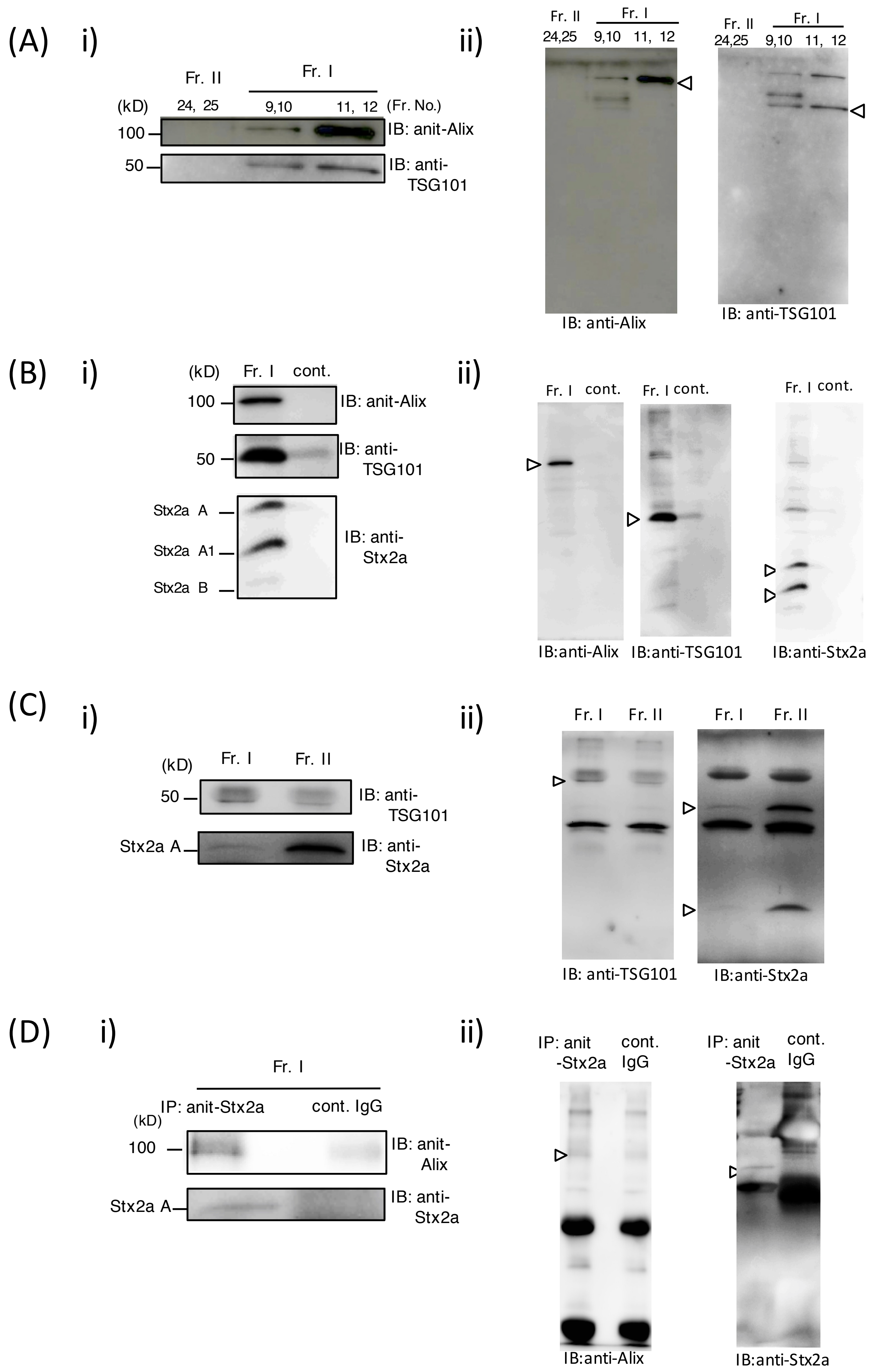


**Supplementary Figure S4. (A-D)** Images presented in i) were cropped from the whole images shown in ii).

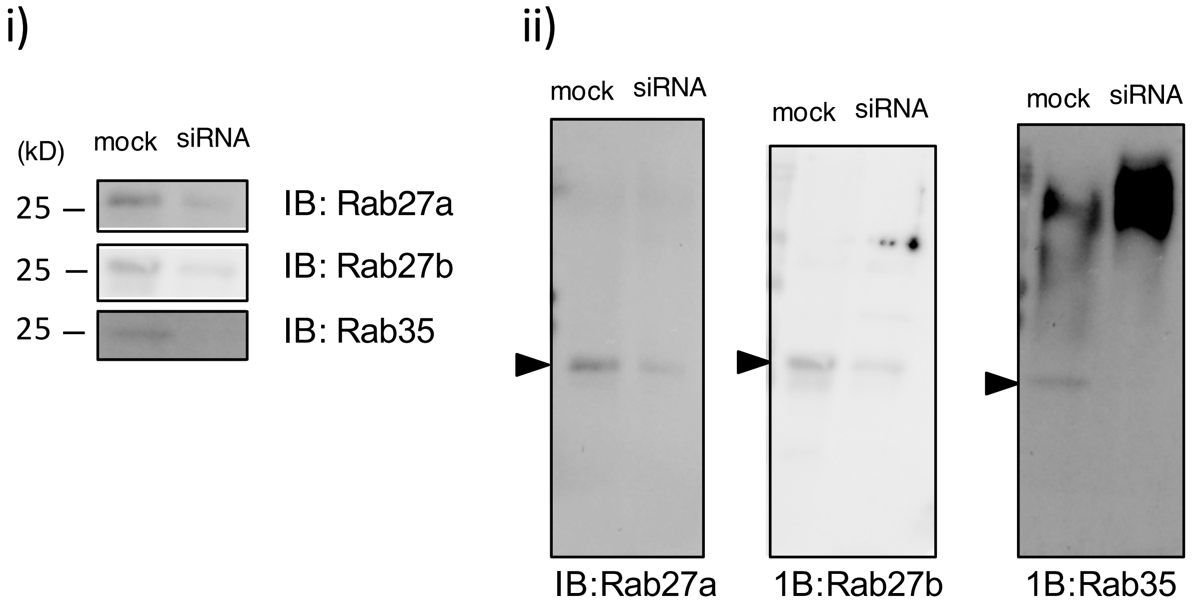


**Supplementary Figure S5.** Images presented in i) were cropped from the whole images shown in ii).
